# Comprehensive Analysis of MGMT Promoter Methylation: Correlation with MGMT Expression and Clinical Response in GBM

**DOI:** 10.1371/journal.pone.0016146

**Published:** 2011-01-07

**Authors:** Nameeta Shah, Biaoyang Lin, Zita Sibenaller, Timothy Ryken, Hwahyung Lee, Jae-Geun Yoon, Steven Rostad, Greg Foltz

**Affiliations:** 1 The Ben and Catherine Ivy Center for Advanced Brain Tumor Treatment, Swedish Neuroscience Institute, Seattle, Washington, United States of America; 2 Department of Radiation and Oncology, University of Iowa Carver College of Medicine, Iowa City, Iowa, United States of America; 3 Iowa Spine and Brain Institute, Waterloo, Iowa, United States of America; 4 Cellnetix Pathology and Laboratories, Seattle, Washington, United States of America; Institute of Cancer Research, United Kingdom

## Abstract

O^6^-methylguanine DNA-methyltransferase (MGMT) promoter methylation has been identified as a potential prognostic marker for glioblastoma patients. The relationship between the exact site of promoter methylation and its effect on gene silencing, and the patient's subsequent response to therapy, is still being defined. The aim of this study was to comprehensively characterize cytosine-guanine (CpG) dinucleotide methylation across the entire MGMT promoter and to correlate individual CpG site methylation patterns to mRNA expression, protein expression, and progression-free survival. To best identify the specific MGMT promoter region most predictive of gene silencing and response to therapy, we determined the methylation status of all 97 CpG sites in the MGMT promoter in tumor samples from 70 GBM patients using quantitative bisulfite sequencing. We next identified the CpG site specific and regional methylation patterns most predictive of gene silencing and improved progression-free survival. Using this data, we propose a new classification scheme utilizing methylation data from across the entire promoter and show that an analysis based on this approach, which we call 3R classification, is predictive of progression-free survival (HR  = 5.23, 95% CI [2.089–13.097], p<0.0001). To adapt this approach to the clinical setting, we used a methylation-specific multiplex ligation-dependent probe amplification (MS-MLPA) test based on the 3R classification and show that this test is both feasible in the clinical setting and predictive of progression free survival (HR  = 3.076, 95% CI [1.301–7.27], p = 0.007). We discuss the potential advantages of a test based on this promoter-wide analysis and compare it to the commonly used methylation-specific PCR test. Further prospective validation of these two methods in a large independent patient cohort will be needed to confirm the added value of promoter wide analysis of MGMT methylation in the clinical setting.

## Introduction

The current standard of care for patients diagnosed with glioblastoma multiforme (GBM) has evolved to include surgery with maximum feasible resection [1], radiotherapy [2] with concomitant temozolomide chemotherapy, followed by adjuvant temozolomide chemotherapy [3]. The addition of temozolomide (TMZ) improved overall and progression-free survival [3], but despite this advance, 70% of patients still experience disease progression within one year [3]. Response to TMZ has been shown to be heterogeneous and debate remains as to the dose and frequency of TMZ administration both in the general patient population as well as in individual patients [4]. Clinical approaches designed to optimize temozolomide effectiveness in individual patients may present an opportunity to extend survival even further [5].

TMZ damages DNA by introducing alkyl groups at multiple sites along the DNA backbone, impairing DNA replication, and triggering cell death. The alkylation of the O^6^ position of guanine is a particularly cytotoxic lesion. Normal cells contain DNA repair proteins that remove these alkyl groups or alkylated nucleotides allowing for normal DNA replication. One of the DNA-repair proteins, O^6^-alkylguanine DNA alkyltransferase (AGT), removes alkyl adducts from the O^6^ position of guanine and the O^4^ position of thymine, effectively restoring these DNA bases and preventing TMZ-induced cell death. The DNA-repair protein AGT is encoded by the gene O^6^-Methlyguanine-DNA-methyltransferase (MGMT). Tumor cells expressing this gene have been shown to be resistant to alkylating agents, while those that lack the DNA-repair protein appear to be more susceptible [6]. In most cases, the silencing of MGMT is associated with methylation of cytosine nucleotides at cytosine-guanine dinucleotides (CpG sites) present in the gene's promoter region.

Although MGMT methylation status appears to be a predictive marker of response to TMZ in patients with newly diagnosed GBM [7], there have been several issues which have prevented widespread adoption of this marker in clinical practice. First, definitive confirmation of the predictive value of MGMT methylation as measured by the commercially available methylation-specific quantitative PCR (qMSP) test (Labcorp, USA) [8] awaits the results of a randomized prospective trial [5]. Second, current techniques available for testing MGMT methylation either lack precision, as is the case with qMSP [9], [10], or are too expensive and time-consuming to be used in clinical practice [11]. Third, the MGMT promoter consists of 97 CpG sites which are not uniformly methylated in individual patients or across a patient population [12]. All previous studies correlating MGMT methylation status with patient response to therapy have tested only a small segment of the downstream promoter [13] whereas methylation of both upstream as well as downstream sites have been shown to repress MGMT transcription [12], [14]. The predictive value of the upstream promoter region has been suggested but not yet correlated with clinical response [12], [14]. Finally, as there are no clear alternative treatments for patients with unfavorable MGMT methylation status, testing has been primarily limited to research trials. Once the predictive value is confirmed and alternative treatments become available [15], it will be important to have a high throughput, standardized diagnostic test to assess MGMT methylation for treatment planning.

Two significant issues need to be addressed in order to optimize MGMT methylation testing for use in clinical practice. First, the specific MGMT promoter regions most predictive of patient response to therapy need to be identified and validated. Second, the heterogeneity of methylation patterns across the promoter region and within individual tumors needs to be characterized in order to optimize probe design for PCR-based methods. To address these issues, we characterized MGMT promoter methylation patterns using bisulfite sequencing to obtain quantitative methylation results for all 97 CpG sites in 70 newly diagnosed GBM patients. Using a similar approach, Everhard et al. have previously shown a correlation between promoter-wide methylation patterns and MGMT mRNA expression [12]. We confirm these findings and further extend this approach to show, for the first time, how individual CpG site-specific methylation patterns correlate with MGMT mRNA expression, MGMT protein immunohistochemistry (IHC), and patient response to primary therapy. We then use statistical modeling to identify the individual CpG sites and related MGMT promoter regions most predictive of TMZ response. Based on this analysis, we propose a new classification scheme that utilizes methylation data from three different regions spanning the entire promoter and show that this approach is predictive of clinical response. Recognizing that bisulfite sequencing is laborious and expensive, we designed a new test utilizing methylation-specific multiplex ligation-dependent probe amplification (MS-MLPA) of individual CpG sites specific to each of the three promoter regions and show that this MS-MLPA based test is both feasible in the clinical setting and potentially more sensitive in identifying patients with MGMT promoter methylation. These findings warrant further prospective clinical study to validate the additional benefit of developing an MS-MLPA based whole promoter test as an alternative or adjunct to the commonly used qMSP test.

## Materials and Methods

### Ethics statement

This study was reviewed and approved by human subjects committees at the University of Iowa IRB (IRB199812055), and Western IRB (IRB00000533) in compliance with the ethical principles as set forth in the report of the National Commission for the Protection of Human Subjects of Biomedical and Behavioral Research entitled “Ethical Principles and Guidelines for the Protection of Human Subjects of Research (Belmont Report)”. The research protocol was also approved by the Swedish Neuroscience Institute research steering committee. All participants provided written informed consent according to IRB guidelines prior to participation in this study.

### Patients and tissue collection

Patient tissue samples were obtained from two institutions, the University of Iowa Hospital and Clinics, IA, USA (Iowa cohort) and Swedish Medical Center, WA, USA (Swedish cohort). All patients gave informed consent prior to collection of specimens according to institutional guidelines. Tissue samples were snap-frozen in the operating room immediately during surgery with an adjacent specimen sent to pathology for diagnosis by a board-certified neuropathologist. Each tumor specimen was archived at −80°C for further DNA, RNA and protein studies. All patients underwent surgery for tumor resection, had post-operative diagnosis of WHO grade IV glioblastoma multiforme (GBM), received radiation therapy with concurrent temozolomide chemotherapy and subsequent temozolomide adjuvant chemotherapy. For the Iowa cohort surgeries were performed between June 1999 to July 2008 and for the Swedish cohort surgeries were performed during the period of February 2007 to April 2010. All clinical data was collected prospectively according to institutional guidelines. Progression free survival (PFS) was calculated as the number of days between the date of surgery and the date of MRI scan that showed disease progression or recurrence.

### Bisulfite sequencing

Genomic DNA was extracted from 25 mg of frozen GBM tissue using ChargeSwitch Genomic DNA mini tissue kit (Invitrogen, Carlsbad,CA). Sodium bisulfite modification of 4 ug was carried out using the MethylEasy DNA bisulfite modification kit (Human Genetic Signatures, Sydney,Australia). The MGMT promoter associated CpG island was identified using the UCSC genome browser [16], [17]. Two rounds of PCR were performed to get 400–500 bp products. The PCR mixture contained 20 pmol of each primer, 20 ng of bisulfite-treated DNA (or 2 ul of first round product), 1.25 units of Taq DNA polymerase (5Prime), 200 mM dNTPs and 1.5 mM Mg(OAc)2 in a final volume of 50 ul. The PCR was performed with initial denaturing at 94°C for 3 min followed by 40 cycles of denaturing at 94°C for 1 min, annealing at 55°C for 2 min, extension for 3 min at 68°C, and a final extension at 68°C for 10 min. The MGMT CpG island is 762 bp in length. It was amplified in two parts. To amplify the region upstream of the transcription start site, the following primer pairs were used: for 1^st^ round, F- AAA ACR AAA TCT AAA ACR AAA CRA AAC CRA AAA CCT AAA AAA AA, R- TAT TAT AGG TTT TGG AGG TTG TTT TTA CGG TTT TTT GAT, and for 2^nd^ round, F- AAA CTA AAA CRA AAC CCR AAA AAA ACR AAA TAT TCC, R- TAG GGT TTT TGT TGG TTT GGG GGT TTT TGA T. To amplify the downstream region, the following primer pairs were used: for 1^st^ round, F- CCA ATC CAC AAT CAC TAC AAC RCA ACC TAA TCC AAA, R- GAA GGG TTA TTY GGG TTA GGY GTA TAG GGT, and for 2^nd^ round, F- CCC AAA CCC GAA AAA AAA CTA AAC AAC ACC TAA AAA A, R- GGY GTT TAG YGA GGA TGY GTA GAT TGT TTT AGG TT. The PCR products were cleaned using ChargeSwith PCR Clean-Up Kit (Invitrogen, Carlsbad,CA) and cloned using the TOPO TA cloning kit(Invitrogen, Carlsbad,CA). The bacterial colonies were inoculated in 96 well plates in 2YT media with appropriate antibiotic. Plasmid DNA were purified using Sprint Prep (Agencourt Biosciences, Beverly,MA) on a Biomek FX(Beckman Coulter). DNA was sequenced using Big Dye Terminator v3.1 (Applied Biosystems, Foster City,CA) and the sequences were resolved on a 3730 XL DNA Analyzer (Applied Biosystems). Multiple clones were sequenced for each patient sample (median of 10 clones). The sequence data was then assembled using the phredPhrap program [18], [19]. BiQ Analyzer software [20] was used for quality control and for calculating fraction methylation at each CpG site.

### MGMT mRNA

Total RNA was isolated from tissue samples with Triazol (Invitrogen), and then cleaned with RNeasy MinElute Cleanup Kit (Qiagen). 1 ug of total RNA was used to generate 100 ul of cDNA using the High Capacity cDNA Reverse Transcription Kit (Applied Biosystems). Real-time PCR of MGMT was performed on the ABI PRISM 7900 HT detection system using a taqman probe (Hs00172470_m1, Applied Biosystems) and taqman reagents under default conditions: 95°C for 10 minutes, 40 cycles of 95°C for 15 seconds, and 60°C for 1 minute with human beta-glucuronidase (hGUS) as endogenous control. All assays were done in triplicate. The expression level of MGMT for each tissue sample was calculated compared to the hGUS expression level using the formula 2^−(Ct value of MGMT – Ct value of hGUS)^.

### MGMT IHC

Tissue sections were fixed in 10% buffered formalin and blocked in paraffin. Sections of 6 micron thickness were incubated with antibodies to O6-methylguanine-DNA methyltransferase (MGMT) from Santa Cruz Biotechnology (MT 3.1/sc-56157). Vizualization of the reaction product was accomplished using a biotinylated secondary antibody exposed to an avidin and biotinylated horseradish peroxidase macromolecular complex. A representative portion of the tumor containing less than 10% necrosis and without evidence of excessive macrophage infiltration was chosen for MGMT IHC studies. The entire immunoslide was reviewed along with the hematoxylin and eosin-stained slide and the percentage of tumor nuclei positive for MGMT was determined. GBM tissue with high MGMT protein expression was used as a positive control. Tissue samples were also assayed with no primary antibody as negative controls to rule out non-specific reaction. Staining of endothelial cells was used as positive internal control. As per Capper et al. (see Discussion section) samples with greater than 15% positively stained nuclei were considered immunopositive for MGMT [21].

### Methylation-Specific Multiplex Ligation-Dependent Probe Amplification (MS-MLPA)

**Figure 1:**
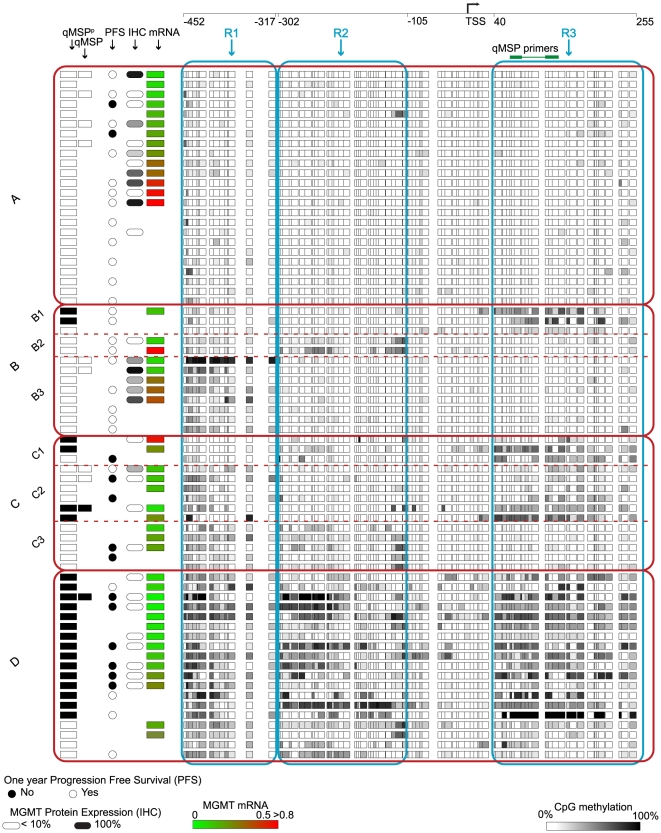
MGMT methylation profiles of 70 GBM samples show four distinct groups. Each row represents an individual patient. For each patient the qMSP^P^ positive result is shown as a black rectangle. The next column shows the results of the qMSP test (Labcorp). PFS is represented by a circle with a blackened circle representing greater than 1 year PFS. Positive MGMT protein expression (>15% tumor cells stained positive in IHC) is shown as black rounded rectangle and negative expression is shown as white rounded rectangle. MGMT mRNA expression is shown using color-coded rectangles. Absence of any of the shapes indicates results not available. All CpG sites belonging to the three regions, R1, R2 and R3 are shown within the blue outlined squares. The four distinct groups are outlined with red squares. The qMSP region is labeled with qMSP primers in R3. The transcription start site (TSS) of the MGMT gene is marked by an arrow.

MLPA probes containing methylation-sensitive HhaI recognition site (GCGC) were annealed to genomic regions (R1, R2, and R3, see Figure 1) of MGMT and ligated. The ligated products were then digested with HhaI, which cuts at unmethylated GCGC sites. After PCR amplification with adapter primers linked to the MLPA-probes, the methylated regions amplify and are detected while the digested unmethylated regions fail to amplify[22]. The SALSA MLPA KIT P200-A1 Human DNA Reference-1 ((MRC-Holland, Amsterdam, The Netherlands) was used to perform the MS-MLPA test. This kit contains control fragments and reference probes. The standard protocol (MS-MLPA DNA Methylation Quantification Protocol, MRC-Holland, Amsterdam, The Netherlands) was followed. For MGMT PCR, F- GGG TTC CCT AAG GGT TGG A, R- GTG CCA GCA AGA TCC AAT CTA GA primers were used. Three sets of primers were used for detecting methylation at CpG site 8 (MLPA-R1), 22 (MLPA-R2) and 80 (MLPA-R3). These CpG sites were chosen based on the presence of the methylation-sensitive HhaI recognition site. CpG 80 also correlated well with mRNA, protein expression and PFS whereas CpG 22 correlated well with mRNA expression and PFS (Table 2). For MLPA-R1, F- GGG TTC CCT AAG GGT TGG ATC AGC GTA GCC GCC CCG AGC A, R- GGA CCG GGA TTC TCA CTA AGC GGG CGC CGT CTC TAG ATT GGA TCT TGC TGG CAC; MLPA-R2, F- GGG TTC CCT AAG GGT TGG AGA CCC CCG CGC GCT TTC AG, R- GAC CAC TCG GGC ACG TGG CAG GTA ATC TAG ATT GGA TCT TGC TGG CAC; and for MLPA-R3, F- GGG TTC CCT AAG GGT TGG AGT CCT CGC GGT GCG CAC CGT T, R- TGC GAC TTG GTG AGT GTC TGG GTC GCC TCG CTC CTC TAG ATT GGA TCT TGC TGG CAC. Three replicates were performed for each sample. Data were analyzed by the Coffalyser software with a cut-off value of 0.3 to identify methylated samples per the manufacturer's recommendation (MRC-Holland). Additionally, the data were also checked by manual analysis.

### Statistical analysis

#### Methylation profile

A methylation profile was calculated for each of the 97 CpG sites in the MGMT promoter associated CpG island in all 70 patient tissue samples. At each CpG site, a fraction of methylation was calculated as the ratio of methylated clones over the total number of clones sequenced per patient tissue sample. A cutoff value of 0.1 was used to allow a binary classification of each site as methylated or unmethylated[12].

#### Stratification of methylation patterns – 3R classification

**Figure 2:**
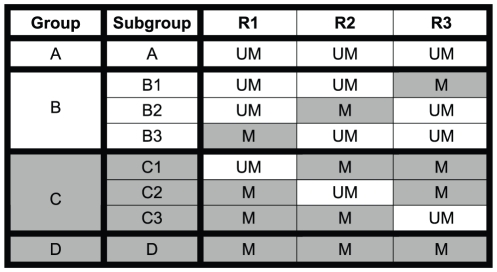
Schematic of 3R classification. All three regions of the MGMT promoter for each patient are classified as methylated (M) or unmethylated (UM). Based on the status of these three regions, patients are classified into four major groups: Group A) all regions unmethylated, Group B) one region methylated, Group C) two regions methylated, and Group D) all three regions methylated. For correlation analysis, Groups B and C were further subdivided as indicated.

A data matrix of methylation profiles from all 70 patients was used as input for the MEV software package [23]. Euclidean distance measure and average link clustering were used to cluster all 97 CpG sites. Three CpG site clusters were identified in which at least one CpG site significantly correlated with mRNA expression, IHC staining, or PFS. CpG sites in each cluster were contiguously located on the CpG island. Three regions were defined based on these three clusters. Region 1 (R1) contains 15 CpG sites, region 2 (R2) contains 29 sites, and region 3 (R3) contains 27 sites (Figure 2). Coordinates from the transcription start site for R1 were −452 to −317, for R2 were −302 to −105 and for R3 were +40 to +255. All three regions of the MGMT promoter for each patient were classified as methylated or unmethylated. The methylation status of each region was determined based on the average methylation of CpG sites in each region with a cutoff value of 0.1. Based on the status of these three regions, patients were classified into four major groups and eight subgroups: Group A - All three regions unmethylated; Group B - one region methylated (B1 = R3 methylated, B2 = R2 methylated, B3 = R1 methylated); Group C - two regions methylated (C1 = R2 and R3 methylated, C2 =  R1 and R3 methylated, C3 =  R1 and R2 methylated); Group D - all regions methylated. These groups are shown in Figure 1. Overall methylation profiles were classified as methylated or unmethylated based on the methylation status of the three regions (3R classification method). If two or more regions were methylated then the profile was classified as methylated (Groups C and D). If less than two regions were methylated then the profile was classified as unmethylated (Groups A and B).

#### Correlation analysis

The correlation of individual CpG site methylation with MGMT mRNA expression level was calculated using Spearman's rank correlation. Fractional methylation and mRNA expression level were both continuous variables. Fractional methylation of a promoter region was calculated by averaging methylation of all CpG sites in that region. For correlation of methylation with IHC staining, binary values were used for both methylation, cutoff set at 0.1 [12], and IHC staining, cutoff set at 15% [21]. Significance of concordance between methylation and IHC was performed using Pearson's chi-square test. The Kaplan–Meier estimator and Hazards Ratio were used to compare PFS distributions between methylated and unmethylated groups. Log rank test was used for p-value calculation. For all analyses, the first six CpG sites were excluded as they are known to be methylated in normal samples [12]. We also excluded CpG sites in the region -99 to 21 because they are unmethylated in more than 80% of the patients precluding the statistical power to achieve significance. The SPSS software package (http://www.spss.com/) was used for this analysis.

#### Predicted Methylation-specific PCR (qMSP^P^)

Results of the commercially-available qMSP test (LabCorp, USA)[8] were only available for ten patients from the Swedish cohort. We sought to predict qMSP results for the rest of the patients by analyzing their bisulfite sequencing data to identify the fraction of methylation for each of the seven CpG sites that span the qMSP test primers. Previous studies which directly compared bisulfite sequencing with MSP for identification of MGMT methylation provided validation for this approach [9], [24]. As seen in 1, the forward primer of the qMSP test spans four CpG sites (forward primer set) and the reverse primer spans three CpG sites (reverse primer set) in region 3 of the MGMT promoter [8]. Based on previously published studies [9], [24] as well as the requirement that each MSP primer must contain more than one CpG site to distinguish between methylated and unmethylated DNA [25], we conservatively estimated that at least two CpG sites from each of the forward and reverse primer sets would need to be methylated in order for the qMSP test to be positive for methylation. We denote this predicted qMSP result as qMSP^P^ to distinguish it from the commercially available qMSP [8]. We also validated our results by comparing our qMSP^P^ to the commercially-available qMSP on ten patient samples for which we found 100% concordant results (1).

## Results

### Sample characteristics

We obtained GBM tissue samples from 70 patients, 45 men (64%) and 25 women (36%) with a median age of 60 years (range, 16–81 years). All were diagnosed with primary GBM with a single lesion, were treatment naïve, underwent surgery for maximal resection and received standard external-beam radiotherapy (59.4 gray) with concomitant TMZ (single daily dose 75 mg/m2) followed by monthly cycles of TMZ chemotherapy (daily dose 200 mg/m2 for five consecutive days)[3].

DNA for assessment of MGMT promoter methylation was available from all 70 patients. MGMT immunohistochemistry (IHC) was performed on 31 patients (Swedish cohort). RNA from 46 patients was available for assessment of MGMT gene expression. Progression-free survival (PFS) data was available for 44 patients out of which 39 patients had at least one year follow-up. Fourteen patients with less than 95% tumor resection were excluded from PFS analysis and twelve patients were lost to follow-up. The Venn diagram in Figure S1 shows how these different measurements overlap in the patient cohorts.

Among the 44 patients with PFS data, the median PFS was 269 days (range, 71–766 days). PFS was not associated with gender but did show a significant association with age where we observed a median PFS of 314 days in younger patients vs 197 days in older patients (HR  = 1.051, 95% CI [1.014–1.091], p = 0.05,). Of note, for the 24 patients in this group for whom IHC results were available, patients with decreased MGMT protein expression (immunonegative, IHC results ≤15%) had significantly better PFS, 540 days vs 197 days, compared to patients with higher MGMT protein expression (immunopositive, IHC results >15%) (HR  = 8.85, 95% CI [2.179–35.714], p<0.0001) (Table 1 and Figure S3). When patient samples were characterized as methylated or unmethylated using the methylation profile of the entire promoter (3R classification, see methods section and described below) there was a strong association between methylation and PFS. The median PFS for the methylated group was 540 days vs. 210 days for the unmethylated group (HR  = 5.23, 95% CI [2.089–13.097], p<0.0001). The multivariate Cox proportional hazard model showed MGMT IHC and 3R classification as independent predictors of PFS (Table 1).

**Table 1:** Sample characteristics and Kaplan-Meier analysis of PFS.

Category	Subcategory	Number of Patients	p-value
**Age**	***<60***	22 (50%)	0.05
	**≥** ***60***	22 (50%)	
**Gender**	***Male***	28 (64%)	0.784
	***Female***	16 (36%)	
* **IHC**	**≤15** ***% (Immunonegative)***	14 (58%)	<0.0001
	***>15% (Immunopositive)***	10 (42%)	
* **3R**	***M***	17 (39%)	<0.0001
	***UM***	27 (61%)	

*The multivariate Cox proportional hazard model showed MGMT IHC and 3R classification as independent predictors of PFS.

### Stratification of methylation patterns into four major groups

High resolution bisulfite sequencing of the entire CpG island in 70 GBM samples revealed highly heterogeneous methylation profiles (Figure 1). Hierarchical clustering of methylation patterns of all 70 GBM samples identified three major sets of CpG site clusters. Each set occupied a contiguous part of the CpG island forming three distinct regions. The coordinates for each region are measured from the transcription start site (TSS). Region 1 (R1) coordinates are −452 to −317, Region 2 (R2) coordinates are −302 to −105, and Region 3 (R3) coordinates are 40 to 255. R1 and R2 are upstream of the TSS whereas R3 is downstream of the TSS. As indicated in Figure 1, the previously reported MSP [26] and qMSP region [8] is contained in R3. The primers used in the commercial qMSP test cover seven CpG sites in R3.

Methylation profile classification based on these three regions led to the identification of four methylation patterns: Group A, the unmethylated group, consisting of samples with all three regions unmethylated; Group B, the lightly methylated group, consisting of samples with only one region methylated; Group C, the moderately methylated group, consisting of samples with any two regions methylated; and Group D, consisting of samples that were densely methylated (Figure 1 and Figure 2).

### Single site-based correlation analysis

Table S1 shows how well methylation at an individual CpG site is correlated with MGMT mRNA expression (46 samples), protein expression (31 samples) and PFS (44 samples). A total of 36 individual CpG sites from across the promoter were found to be significantly correlated with MGMT mRNA expression. Of note, six of these CpG sites are located in the region measured by the qMSP test (Labcorp, USA)[8]. We found that fewer CpG sites, 25 total, were significantly correlated with MGMT protein expression (p<0.05, Pearson's chi-square test). The highest concordance between an individual CpG site methylation and protein expression was 71%. Four of the seven sites within the qMSP test region were significantly correlated with protein expression. A total of 17 CpG sites were found to be predictive of PFS. Two of them were located in R1, seven in R2 and eight in R3. Only one of the seven sites of the qMSP test (LabCorp, USA)[8] was significantly correlated with PFS. Of note, seven CpG sites, CpG 38, CpG 73, CpG 80, CpG 81, CpG 82, CpG 86 and CpG 89 (Table 2), were found to be significantly correlated across all three variables: mRNA expression, protein expression and PFS.

**Table 2:** Significant individual CpG site correlations with MGMT mRNA expression, MGMT protein expression and PFS.

CpG site	distance from TSS	%Meth	Region	mRNA (ρ)	Protein (%concordance)	PFS (hazard ratio [95% confidence interval])
8	−448	78	R1, MLPA-R1	−0.159	61	1.881 [0.868–4.076]
22	−302	35	R2, MLPA-R2	−.407**	58	2.460 [1.066–5.674]*
38#	−181	33	R2	−.371*	68**	2.463 [0.995–6.098]*
73#	53	35	R3	−.362*	65**	3.084 [1.063–8.948]*
75	65	35	R3, qMSP	−.376**	68**	1.590 [0.678–3.73]
76	68	28	R3, qMSP	−.452**	58*	1.688 [0.687–4.151]
77	72	28	R3, qMSP	−.453**	58	2.153 [0.876–5.292]
78	82	26	R3, qMSP	−.384**	52	1.186 [0.483–2.916]
80#	89	43	R3, MLPA-R3	−.408**	68*	2.311 [1.021–5.23]*
81#	94	37	R3	−.292*	68*	2.539 [1.076–5.992]*
82#	100	37	R3	−.321*	68**	2.508 [1.102–5.711]*
86#	142	39	R3	−.399**	68*	2.903 [1.17–7.2]*
87	155	43	R3, qMSP	−.334*	68*	1.786 [0.793–4.025]
88	160	24	R3, qMSP	−0.162	52	1.837 [0.638–5.289]
89#	172	54	R3, qMSP	−.338*	71*	2.255 [1.071–4.748]*

**ρ** = Spearman's rank correlation coefficient.

*p-value <0.05.

**p-value <0.01.

# These seven CpG sites are significantly correlated with all three measures: MGMT mRNA expression, protein expression and progression-free survival.

### Region-based correlation analysis

Methylation of any of the three major CpG cluster regions was significantly correlated with mRNA expression (p<0.05). The methylation status of regions R2 and R3, but not R1, showed significant concordance with protein expression and PFS (Table 3).

**Table 3:** Correlation of MGMT promoter methylation patterns with MGMT mRNA expression, MGMT protein expression and PFS.

Classification	mRNA (ρ)	Protein (%concordance)	PFS (hazard ratio [95% confidence interval])
R1	−.382**	55	2.034 [0.964–4.29]
R2	−.373*	74**	2.275 [1.025–5.052]*
R3	−.321*	71**	2.710 [1.243–5.908]**
3R	−.424**	74**	5.230 [2.089–13.097]**
MLPA	−.545**	71**	3.076 [1.301–7.27]**
qMSP	−.459**	71**	1.707 [0.728–4.003]

**ρ** =  Spearman's rank correlation coefficient.

*p-value <0.05.

**p-value <0.01.

When analyzed by groups, the largely unmethylated group A consisted of a subset of samples with high mRNA expression (student's t test, p>0.05) and high protein expression (p = 0.02) when compared to Groups C and D. Twelve out of fourteen patients had disease progression before one year (HR 5.23, 95% CI [2.089–13.097], p<0.0001, median PFS of 540 days in Groups C and D vs 210 days in Group A). In contrast, Group D, the highly methylated group in which all three regions are methylated compared to Groups A and B, had low mRNA expression (student's t test, p = 0.036) and negative protein expression (p = 0.001). Six out of the 10 patients in this group had PFS greater than one year (HR  = 3.312, 95% CI [1.192–9.201], p = 0.016, median PFS of 373 days in Group D vs 210 days in Groups A and B).

Group B, the lightly methylated group consisting of samples with one out of three regions methylated, was similar to Group A; consisting of a subset of samples with high protein expression (p = 0.04) and high mRNA expression approaching statistical significance (student's t test, p = 0.07). Nine out of nine patients had disease progression before one year (HR  = 8.906, 95% CI [2.244–35.343], p<0.0001, median PFS of 540 days in Groups C and D vs 202 days in Group B). Group C, the moderately methylated group consisting of samples with any two regions methylated, was similar to Group D. The samples in this group compared to groups A and B had low mRNA expression (student's t test, p>0.05) and negative protein expression (p = 0.006). Five out of six patients had over one year progression-free-survival (HR  = 10.002, 95% CI [2.214–45.179], p = 0.003, median PFS of 753 days in Group C vs 210 days in Groups A and B). Subgroup analysis of Groups B and C did not reveal any significant difference in correlation with mRNA expression, protein expression or PFS within each subgroup compared with the parent group.

**Figure 3:**
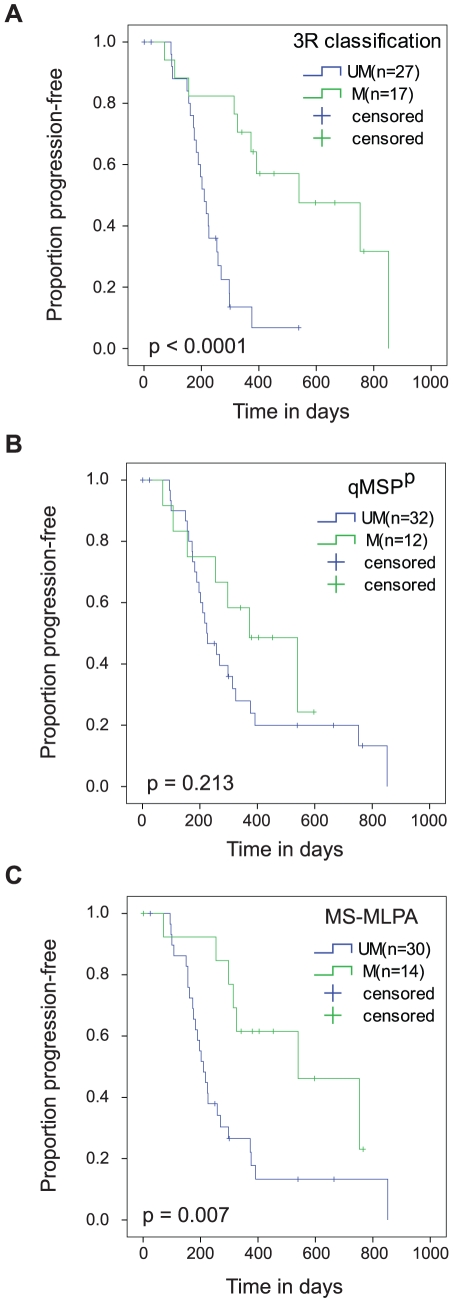
3R classification and MS-MLPA predict PFS in GBM. Kaplan-Meier estimation of PFS according to MGMT promoter methylation status determined using: A) 3R classification method - The median PFS for the methylated group was 540 days vs. 210 days for the unmethylated group (HR  = 5.23, 95% CI [2.089–13.097], p<0.0001), B) qMSP^ P^ - The median PFS for the methylated group was 373 days vs. 224 days for the unmethylated group (HR  = 1.707, 95% CI [0.728–4.003], p = 0.213), and C) MS-MLPA - The median PFS for the methylated group was 540 days vs. 210 days for the unmethylated group (HR  = 3.076, 95% CI [1.301–7.27], p = 0.007).

We classified Group C and Group D samples as methylated and Group A and B samples as unmethylated. This classification of methylation patterns (3R classification) showed strong correlation with mRNA expression and protein expression (Table 3 and Table S2). The methylated group identified by the 3R classification also correlated with longer PFS (Table 3, Figure 3A). Of note, methylation patterns in the qMSP region failed to correlate with progression-free survival in our cohort (Table 3, Figure 3B) but did show correlation with mRNA expression and protein expression.

### MS-MLPA

Analysis of the entire MGMT promoter suggested that a test investigating methylation of individual CpG sites across the entire promoter might be more informative than methods focused on a single region. We recognize that bisulfite sequencing for 3R classification is not feasible in the clinical setting as it is an expensive and time consuming method that cannot be performed on paraffin-fixed or archived tissue. To overcome these limitations, we used an MS-MLPA based test [22] which can detect MGMT methylation in all three promoter regions at the key CpG sites used in the 3R classification. Three different MS-MLPA assays were developed: 1) MLPA-R1 assessed CpG 8 methylation in region R1, 2) MLPA-R2 assessed CpG 22 methylation in region R2, and 3) MLPA-R3 assessed CpG 80 methylation in region R3. We obtained MS-MLPA results for all 70 patients for which bisulfite sequencing data were available. We then classified samples with two or more positive MS-MLPA probes into the methylated group and assigned the rest into the unmethylated group. This MS-MLPA based 3R classification showed high correlation with mRNA expression, protein expression and progression-free survival (p<0.01, see Table 3 and Figure 3C).

### MS-MLPA and qMSP

**Figure 4:**
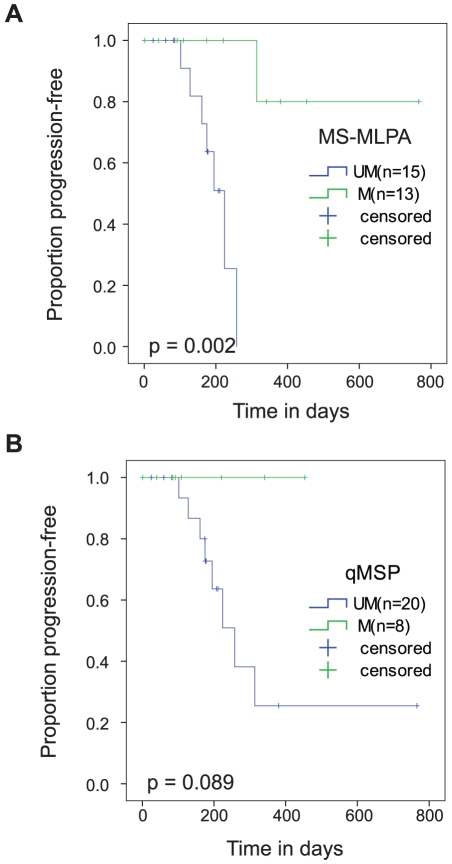
MS-MLPA predicts PFS in 28 GBM patients. Kaplan-Meier estimation of PFS according to MGMT promoter methylation status determined using: A. MS-MLPA (p-value  = 0.002) and B. qMSP (p-value  = 0.089).

Results of the commercially available qMSP test (Labcorp, USA) [8] were available for 28 patients in the Swedish cohort. In these 28 patients, the MS-MLPA based 3R classification test identified 13 patients (46%) as having MGMT methylation compared to only 8 (29%) patients identified by the qMSP test. Kaplan-Meier analysis for PFS showed that MS-MLPA classification successfully differentiated methylated patients with improved PFS whereas the qMSP test had not yet achieved significance (see Figure 4), with the caveat that the majority of these patients in this small cohort had a follow-up of less than one year. We examined three patients who were identified as unmethylated by qMSP but methylated by MS-MLPA classification and had a follow-up of more than one year. One of these patients had 314 days of progression-free survival whereas the other two patients remain progression-free at 380 and 766 days. Thus, in this small cohort of 28 patients, it is interesting to observe that MLPA classification appears to be more sensitive in identifying patients with clinically significant MGMT methylation who might otherwise be missed using the qMSP test. These observations need to be confirmed in a larger prospective study.

## Discussion

In this study, we sought to further define the relationship between MGMT promoter methylation, mRNA expression, protein IHC and clinical response to therapy. We chose a uniform patient population who shared the same diagnosis and underwent similar treatment regimens. We used progression-free-survival as the clinical outcome measure in order to avoid differences between patients in response to post-progression treatment. This endpoint has been recently validated by a study which showed that six-month progression-free survival can be used as an alternative primary efficacy endpoint to overall survival in newly diagnosed glioblastoma patients receiving temozolomide [27]. We used bisulfite sequencing, which is currently the most comprehensive technology available to characterize methylation patterns across the whole MGMT promoter, and, because it is quantitative, allowed us to stratify patients in four major groups with varying levels of methylation instead of using a binary classification.

In general, we observed that methylation profiles in GBM patients are heterogeneous across the promoter region and also within individual clones from the same tissue sample, most likely representing the heterogeneity of cell types within the tumor sample (Figure S2). Methylation of either upstream (R1, R2) or downstream (R3) regions resulted in transcriptional repression and decreased protein expression with the exception of one patient (in subgroup C2) that showed 30% MGMT positive cells. Although methylation of either of the downstream or upstream region was sufficient for transcriptional repression, improved response to treatment as measured by PFS was observed only for Group C and Group D where a larger part of the promoter region was methylated.

Our findings with respect to the effect of MGMT methylation on MGMT transcription are consistent with those of an earlier study in cell lines [14], in which MGMT transcription was shown to be regulated by methylation of both upstream and downstream promoter regions. In the cell lines, methylation of either the upstream or downstream promoter down-regulated mRNA expression with downstream methylation being almost as effective as methylation of the entire promoter. In a recent study [12], Everhard et al. also studied methylation across the entire promoter region of 70 GBM patients and showed that the majority of CpG sites correlated with mRNA expression. In that study, patients were classified into three groups: methylated, unmethylated and intermediate methylation. The unmethylated group had the highest mRNA expression, intermediate group had lower expression and the methylated group had the lowest. Both upstream and downstream promoter regions appeared to have an additive effect on mRNA expression. Our results confirm and extend these findings to include correlation of methylation at individual CpG sites to protein expression and progression free survival. In addition, we show that the three individual CpG sites (CpG 72, CpG 74 and CpG 78) identified by Everhard et al. as most predictive of mRNA expression fail to correlate significantly with PFS in our cohort.

MGMT methylation assessed by the qMSP test has been consistently shown to predict response to the alkylating agent TMZ, measured by either PFS or overall survival, in GBM patients [13], [28], [29], [30], [31], [32]. The question remains as to whether there is any additional benefit, either in sensitivity or predictive value, from measuring methylation in upstream promoter regions. Methylation of the upstream MGMT promoter region has been shown to repress transcription [12], [14], [33] but its value for the purposes of patient prognosis has not yet been explored. In our cohort, the downstream region qMSP^P^ methylation status, although not significantly correlated with PFS, did show a lower frequency of disease progression in the methylated group (45% vs 75% at 1-year follow-up). We suspect that this would trend toward statistical significance with a larger patient population. Of note, when the methylation status was determined using our 3R method, the frequency of disease progression was 31% in the methylated group vs. 91% in the unmethylated group, which achieved statistical significance within the study cohort. These results show for the first time that the upstream region is potentially important in considering the design and development of a clinical test to measure MGMT methylation.

The MGMT methylation patterns observed in this study confirm that the downstream region interrogated by the qMSP test is well chosen given the constraint that only one region is assessed. But our data also suggest that a semi-quantitative test that can measure methylation across the entire promoter and is not dependent on co-methylation of multiple sites may be more informative than qMSP and other MSP-based tests that measure methylation only at the single downstream region and appear to fail when the CpG sites spanning MSP primers are not densely methylated (See Figure 2 – group C2). Unfortunately, bisulfite sequencing is laborious, costly, and requires fresh frozen tissue, which makes it an unrealistic method for routine diagnostics in the clinical setting. In contrast, MS-MLPA allows interrogation of individual CpG site methylation across the entire promoter region and, as a PCR-based test, is more readily adapted to the clinical setting. With that rationale, we utilized an MS-MLPA [22] test for 3R classification in which the three CpG sites interrogated by the MS-MLPA probes are derived from the three different promoter regions which are used for the 3R classification. Our MS-MLPA based 3R classification showed excellent correlation to mRNA and protein expression. The methylated group by MS-MLPA 3R classification was highly associated with improved progression-free survival (58% were progression-free at one year in the methylated group vs. 22% in the unmethylated group). A comparison of the MS-MLPA 3R test with the qMSP test (LabCorp, USA) [8] in a 28 patient cohort showed it was potentially more sensitive in identifying methylated patients (46% methylated patients vs. 29%). Our results suggest that further analysis in a larger independent patient cohort would further define the potential advantages of a whole promoter MS-MLPA based analysis.

There is an ongoing debate about whether MGMT IHC is a good prognostic or predictive marker in GBM. Even though the majority of studies have shown MGMT IHC does not correlate with patient survival [24], [29], [30], [34], Capper et al. showed that if the test is done before radiation and chemotherapy, if evaluation is done with special attention to the infiltration zone of diffuse astrocytomas, and if glioma grade is taken into account for cutoff values, then an IHC result of greater than 15% positively stained nuclei can indeed predict survival in GBM patients [21]. All of the large-scale studies to date [30], [34], [35] have failed to find any concordance between MGMT IHC results and promoter methylation as measured by the MSP test; although a couple of small studies have shown concordance [36], [37]. The observed concordance between our MGMT IHC results and promoter methylation status as defined by 3R classification might be attributed to the fact that we are taking into consideration the methylation status of the entire promoter which may be a better predictor of protein expression than the MSP test which is restricted to a small segment of Region 3. Of note, the combination of IHC with the MSP test has been used to identify a methylated-immunonegative group with a more positive prognosis [30]. In our subset of patients in which IHC results were available, we observed a strong correlation between the immunopositive group and reduced PFS. A subset of patients, those that had no MGMT protein expression despite the fact they had no detectable promoter methylation, are of particular interest. It is difficult to say whether the gene requires activation which may be triggered by temozolomide treatment [38], [39], the gene is silenced by other mechanisms [40], [41], or if it is due to tumor heterogeneity [30], [42]. This patient group with unmethylated-immunonegative MGMT status has been shown to have the worst prognosis [30]. In our study three out of four patients falling in this group had less than one year PFS.

Our findings suggest that careful consideration should be given to single CpG site evaluation in the development of clinical tests to measure methylation given the degree of heterogeneity seen in methylation patterns both within an individual patient and across the patient population. A whole promoter method focused on key single CpG site predictors of response to therapy might have an advantage over semi-quantitative approaches focused on a single region. We anticipate that further refinements in a MGMT methylation test for use in clinical decision-making might include a test similar to our MS-MLPA 3R classification method. Further validation of this approach will require a prospective study to show how methylation of the entire MGMT promoter region affects MGMT mRNA and protein expression and whether it can reliably improve upon current methodology to predict patient response to therapy.

## Supporting Information

Figure S1 **Clinical measurements.** This venn diagram shows the overlap in our patient population of measurements of MGMT mRNA, MGMT protein and progression-free survival. Seventy patient samples were assessed for MGMT promoter methylation. MGMT protein assessment was available for 31 patients. MGMT gene expression could be assessed for 46 of the patients. Radiology reports for assessment of one year PFS were available for 39 patients.
(DOC)

Figure S2 **Clonal heterogeneity within an individual tumor sample.** Each line represents a single clone sequenced from an individual patient's tumor sample. Each circle represents a single CpG site with the filled circles indicating that the site is methylated. For an individual tumor sample, the fractional methylation at a single CpG site is calculated by dividing the number of positive methylated clones by the total number of clones sequenced. In this patient, a total of 24 clones were sequenced.
(DOC)

Figure S3 **Age and MGMT protein predict PFS in GBM.** Kaplan-Meier estimation of PFS determined using: A) Age - The median PFS in younger patients was 314 days vs 197 days in older patients (HR  = 1.051, 95% CI [1.014–1.091], p = 0.05). B) Gender - The median PFS for the females was 224 days vs. 258 days for male patients (HR  = 0.902, 95% CI [0.432 – 1.885], p = 0.784), and C) MGMT protein (IHC) – The median PFS of patients with decreased MGMT protein expression (immunonegative, IHC results ≤15%) was significantly better, 540 days vs 197 days, compared to patients with higher MGMT protein expression (immunopositive, IHC results >15%) (HR  = 8.85, 95% CI [2.179–35.714], p<0.0001).
(DOC)

Table S1 **Single site correlation.**
(DOC)

Table S2 **Correlation of MGMT promoter methylation patterns with MGMT mRNA expression, MGMT protein expression and PFS.**
(DOC)
