# Supplementary material for: Comprehensive Analysis of MGMT Promoter Methylation: Correlation with MGMT Expression and Clinical Response in GBM
Source: PLoS One. 2011 Jan 7;6(1):e16146. doi: 10.1371/journal.pone.0016146 (PMC3017549; doi:10.1371/journal.pone.0016146)
Supplement: Figure S1 — Clinical measurements. This venn diagram shows the overlap in our patient population of measurements of MGMT mRNA, MGMT protein and progression-free survival. Seventy patient samples were assessed for MGMT promoter methylation. MGMT protein assessment was available for 31 patients. MGMT gene expression could be assessed for 46 of the patients. Radiology reports for assessment of one year PFS were available for 39 patients. (DOC) [file pone.0016146.s001.doc]

Figure S1 Clinical measurements.

.

24

16

12

4

6

1

PFS (44)

1 yr PFS (39)

Protein (31)

mRNA (46)

MGMT promoter bisulfite sequencing (70)
