# Supplementary figures and images for: Comprehensive Analysis of MGMT Promoter Methylation: Correlation with MGMT Expression and Clinical Response in GBM
Source: PLoS One. 2011 Jan 7;6(1):e16146. doi: 10.1371/journal.pone.0016146 (PMC3017549; doi:10.1371/journal.pone.0016146)

Figure S2 Clonal heterogeneity within an individual tumor sample.


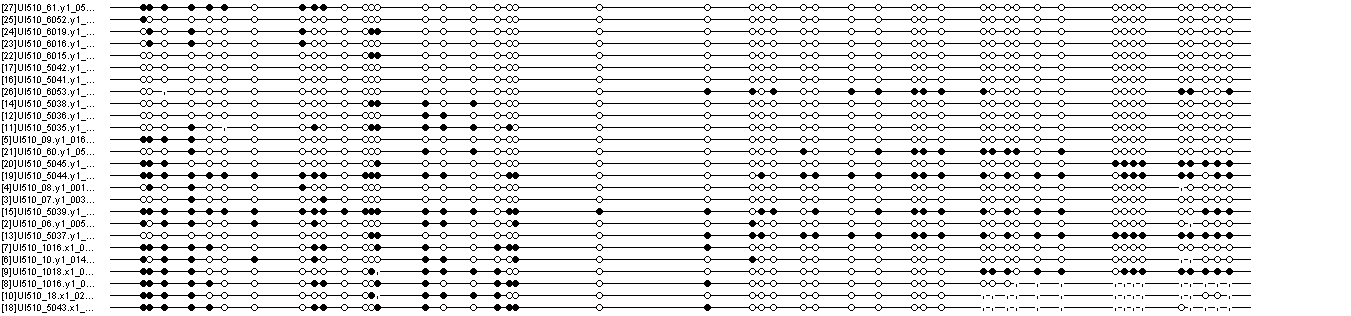

Supplement: Figure S2 — Clonal heterogeneity within an individual tumor sample. Each line represents a single clone sequenced from an individual patient's tumor sample. Each circle represents a single CpG site with the filled circles indicating that the site is methylated. For an individual tumor sample, the fractional methylation at a single CpG site is calculated by dividing the number of positive methylated clones by the total number of clones sequenced. In this patient, a total of 24 clones were sequenced. (DOC) [file pone.0016146.s002.doc]

Figure S3 Age and MGMT protein predict PFS in GBM.


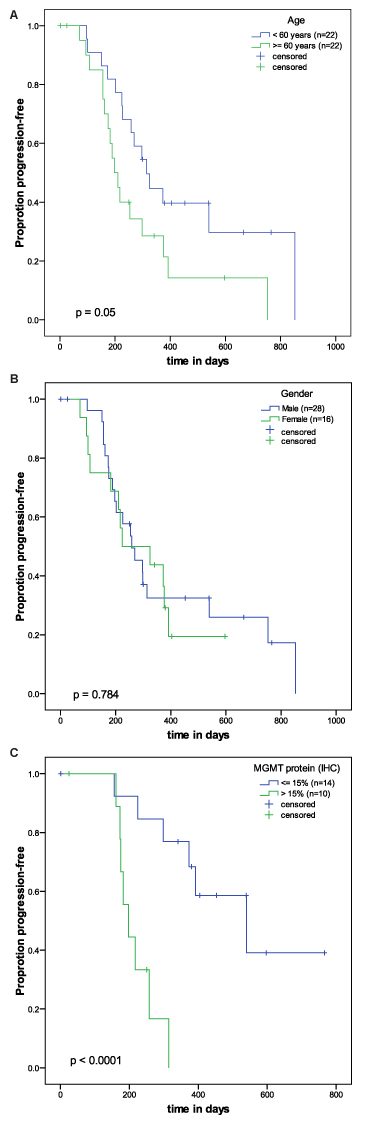

Supplement: Figure S3 — Age and MGMT protein predict PFS in GBM. Kaplan-Meier estimation of PFS determined using: A) Age - The median PFS in younger patients was 314 days vs 197 days in older patients (HR = 1.051, 95% CI [1.014–1.091], p = 0.05). B) Gender - The median PFS for the females was 224 days vs. 258 days for male patients (HR = 0.902, 95% CI [0.432 – 1.885], p = 0.784), and C) MGMT protein (IHC) – The median PFS of patients with decreased MGMT protein expression (immunonegative, IHC results ≤15%) was significantly better, 540 days vs 197 days, compared to patients with higher MGMT protein expression (immunopositive, IHC results >15%) (HR = 8.85, 95% CI [2.179–35.714], p<0.0001). (DOC) [file pone.0016146.s003.doc]
