# Supplementary material for: Comprehensive Analysis of MGMT Promoter Methylation: Correlation with MGMT Expression and Clinical Response in GBM
Source: PLoS One. 2011 Jan 7;6(1):e16146. doi: 10.1371/journal.pone.0016146 (PMC3017549; doi:10.1371/journal.pone.0016146)
Supplement: Table S1 — Single site correlation. (DOC) [file pone.0016146.s004.doc]

Table S1 Single site correlation

| **CpG site** | **distance from TSS** | **%Meth** | **Test** | **mRNA ρ, p-value** | **Protein %concordance, p-value chi square** | **PFS hazard ratio; 95% confidence interval; Median ratio, Log Rank:Mantel-Cox p-value** |
| --- | --- | --- | --- | --- | --- | --- |
| 1 | -503 | 96 | NA | NA | NA | NA |
| 2 | -498 | 91 | NA | NA | NA | NA |
| 3 | -489 | 100 | NA | NA | NA | NA |
| 4 | -483 | 98 | NA | NA | NA | NA |
| 5 | -478 | 87 | NA | NA | NA | NA |
| 6 | -468 | 80 | NA | NA | NA | NA |
| 7 | -452 | 52 | R1 | -.319*, 0.031 | 65, 0.106 | [2.094; 0.997-4.398], 1.44, 0.046* |
| 8 | -448 | 78 | R1, MLPA-A | -0.159, 0.292 | 61, 0.447 | [1.881; 0.868-4.076], 1.45, 0.103 |
| 9 | -445 | 65 | R1 | -0.258, 0.083 | 71, 0.035* | [2.254; 1.093-4.646], 1.5, 0.024* |
| 10 | -438 | 48 | R1 | -.348*, 0.018 | 58, 0.293 | [1.598; 0.774-3.3], 1.45, 0.201 |
| 11 | -431 | 48 | R1 | -.416**, 0.004 | 71, 0.018* | [1.502; 0.711-3.173], 1.47, 0.282 |
| 12 | -429 | 43 | R1 | -0.28, 0.06 | 55, 0.347 | [1.138; 0.555-2.331], 0.98, 0.724 |
| 13 | -427 | 57 | R1 | -.466**, 0.001 | 58, 0.379 | [1.774; 0.845-3.725], 1.5, 0.125 |
| 14 | -411 | 61 | R1 | -.457**, 0.001 | 55, 0.788 | [1.494; 0.715-3.125], 1.39, 0.283 |
| 15 | -405 | 50 | R1 | -0.242, 0.105 | 55, 0.552 | [1.074; 0.522-2.208], 1.02, 0.846 |
| 16 | -395 | 28 | R1 | -0.238, 0.111 | 45, 0.763 | [1.366; 0.558-3.345], 0.78, 0.492 |
| 17 | -387 | 48 | R1 | -0.251, 0.092 | 58, 0.552 | [1.181; 0.577-2.419], 1.24, 0.648 |
| 18 | -383 | 30 | R1 | -0.049, 0.745 | 39, 0.447 | [1.762; 0.72-4.315], 1.45, 0.209 |
| 19 | -381 | 28 | R1 | -0.124, 0.413 | 42, 0.798 | [1.428; 0.546-3.739], 1.44, 0.465 |
| 20 | -353 | 50 | R1 | -0.164, 0.277 | 48, 0.981 | [1.127; 0.544-2.337], 1.24, 0.747 |
| 21 | -317 | 30 | R1 | -0.051, 0.735 | 52, 0.492 | [1.362; 0.599-3.098], 0.98, 0.459 |
| 22 | -302 | 35 | R2, MLPA-B | -.407**, 0.005 | 58, 0.077 | [2.46; 1.066-5.674], 2.41, 0.03* |
| 23 | -299 | 35 | R2 | -0.279, 0.06 | 61, 0.017* | [3.337; 1.336-8.336], 3.47, 0.007** |
| 24 | -295 | 20 | R2 | -.305*, 0.039 | 55, 0.052 | [1.807; 0.625-5.221], 2.13, 0.267 |
| 25 | -285 | 20 | R2 | -.328*, 0.026 | 55, 0.052 | [5.214; 1.233-22.04], NA, 0.012* |
| 26 | -281 | 30 | R2 | -0.218, 0.145 | 68, 0.005** | [3.544; 1.06-11.848], 2.41, 0.029* |
| 27 | -269 | 28 | R2 | -.454**, 0.001 | 58, 0.077 | [2.829; 1.063-7.532], 2.41, 0.03* |
| 28 | -260 | 26 | R2 | -.378**, 0.001 | 52, 0.089 | [2.641; 0.796-8.763], 2.41, 0.099 |
| 29 | -248 | 37 | R2 | -.427**, 0.003 | 61, 0.044* | [1.092; 0.498-2.392], 1.17, 0.826 |
| 30 | -245 | 33 | R2 | -.377**, 0.001 | 58, 0.077 | [1.697; 0.723-3.982], 1.72, 0.219 |
| 31 | -239 | 35 | R2 | -0.22, 0.143 | 58, 0.077 | [1.947; 0.83-4.565], 1.81, 0.119 |
| 32 | -225 | 37 | R2 | -.404**, 0.005 | 68, 0.005** | [2.193; 0.889-5.41], 1.81, 0.08 |
| 33 | -222 | 30 | R2 | -.364*, 0.013 | 61, 0.017* | [2.119; 0.855-5.247], 1.81, 0.097 |
| 34 | -217 | 15 | R2 | -0.177, 0.238 | 58, 0.03* | [1.633; 0.622-4.284], 1.81, 0.314 |
| 35 | -214 | 28 | R2 | -.454**, 0.001 | 65, 0.009** | [1.381; 0.61-3.128], 1.28, 0.437 |
| 36 | -207 | 24 | R2 | -.323*, 0.029 | 55, 0.132 | [1.345; 0.574-3.152], 1.28, 0.493 |
| 37 | -199 | 20 | R2 | -0.275, 0.065 | 58, 0.03* | [5.338; 1.255-22.705], NA, 0.012* |
| 38 | -181 | 33 | R2 | -.371*, 0.011 | 68, 0.005** | [2.463; 0.995-6.098], 2.41, 0.044* |
| 39 | -178 | 11 | R2 | -0.221, 0.139 | 52, 0.089 | [1.344; 0.402-4.494], 1.47, 0.63 |
| 40 | -175 | 9 | R2 | -0.013, 0.931 | 48, 0.148 | [1.353; 0.319-5.747], 1.54, 0.68 |
| 41 | -172 | 20 | R2 | -.321*, 0.03 | 52, 0.089 | [0.78; 0.317-1.92], 1.26, 0.588 |
| 42 | -159 | 20 | R2 | -.451**, 0.002 | 52, 0.089 | [0.673; 0.254-1.781], 0.58, 0.422 |
| 43 | -156 | 26 | R2 | -0.219, 0.143 | 52, 0.123 | [0.587; 0.251-1.369], 0.84, 0.212 |
| 44 | -151 | 20 | R2 | -0.23, 0.124 | 48, 0.148 | [1.194; 0.414-3.444], 1.45, 0.742 |
| 45 | -147 | 9 | R2 | -0.075, 0.622 | NA, NA | [0.187; 0.023-1.517], 0.58, 0.077 |
| 46 | -143 | 9 | R2 | -0.112, 0.458 | 42, 0.419 | [0.802; 0.189-3.406], 0.58, 0.764 |
| 47 | -132 | 15 | R2 | -0.172, 0.252 | 42, 0.419 | [0.865; 0.205-3.652], 0.84, 0.844 |
| 48 | -123 | 30 | R2 | -0.154, 0.307 | 58, 0.03* | [3.212; 0.971-10.627], 3.8, 0.043* |
| 49 | -116 | 33 | R2 | -0.135, 0.371 | 55, 0.052 | [2.179; 0.83-5.721], 1.81, 0.105 |
| 50 | -105 | 33 | R2 | -0.161, 0.286 | 58, 0.03* | [1.864; 0.712-4.882], 1.75, 0.197 |
| 51 | -99 | 7 | NA | NA | NA | NA |
| 52 | -97 | 0 | NA | NA | NA | NA |
| 53 | -95 | 9 | NA | NA | NA | NA |
| 54 | -90 | 15 | NA | NA | NA | NA |
| 55 | -84 | 9 | NA | NA | NA | NA |
| 56 | -78 | 7 | NA | NA | NA | NA |
| 57 | -76 | 13 | NA | NA | NA | NA |
| 58 | -74 | 13 | NA | NA | NA | NA |
| 59 | -49 | 11 | NA | NA | NA | NA |
| 60 | -43 | 7 | NA | NA | NA | NA |
| 61 | -38 | 13 | NA | NA | NA | NA |
| 62 | -26 | 9 | NA | NA | NA | NA |
| 63 | -19 | 7 | NA | NA | NA | NA |
| 64 | -16 | 7 | NA | NA | NA | NA |
| 65 | -7 | 9 | NA | NA | NA | NA |
| 66 | 2 | 7 | NA | NA | NA | NA |
| 67 | 8 | 11 | NA | NA | NA | NA |
| 68 | 14 | 4 | NA | NA | NA | NA |
| 69 | 16 | 11 | NA | NA | NA | NA |
| 70 | 21 | 13 | NA | NA | NA | NA |
| 71 | 40 | 26 | R3 | -0.163, 0.278 | 48, 0.148 | [0.903; 0.344-2.371], 0.88, 0.835 |
| 72 | 42 | 35 | R3 | -.337*, 0.022 | 61, 0.044* | [2.478; 0.939-6.539], 2.41, 0.058 |
| 73 | 53 | 35 | R3 | -.362*, 0.013 | 65, 0.009** | [3.084; 1.063-8.948], 2.41, 0.029* |
| 74 | 60 | 28 | R3 | -.332*, 0.024 | 58, 0.03* | [3.547; 0.841-14.97], NA, 0.066 |
| 75 | 65 | 35 | R3, qMSP | -.376**, 0.001 | 68, 0.005** | [1.59; 0.678-3.73], 1.67, 0.282 |
| 76 | 68 | 28 | R3, qMSP | -.452**, 0.001 | 58, 0.039* | [1.688; 0.687-4.151], 1.45, 0.248 |
| 77 | 72 | 28 | R3, qMSP | -.453**, 0.002 | 58, 0.086 | [2.153; 0.876-5.292], 2.41, 0.087 |
| 78 | 82 | 26 | R3, qMSP | -.384**, 0.008 | 52, 0.226 | [1.186; 0.483-2.916], 1.28, 0.709 |
| 79 | 84 | 37 | R3 | -.376**, 0.001 | 58, 0.077 | [1.101; 0.501-2.419], 1.4, 0.81 |
| 80 | 89 | 43 | R3, MLPA-C | -.408**, 0.005 | 68, 0.012* | [2.311; 1.021-5.23], 1.78, 0.039* |
| 81 | 94 | 37 | R3 | -.292*, 0.0486 | 68, 0.012* | [2.539; 1.076-5.992], 2.57, 0.028* |
| 82 | 100 | 37 | R3 | -.321*, 0.03 | 68, 0.005** | [2.508; 1.102-5.711], 2.57, 0.024* |
| 83 | 121 | 46 | R3 | -.371*, 0.011 | 61, 0.082 | [1.872; 0.884-3.961], 1.72, 0.096 |
| 84 | 126 | 33 | R3 | -.300*, 0.043 | 55, 0.132 | [2.915; 1.171-7.252], 3.47, 0.017* |
| 85 | 132 | 30 | R3 | -.476**, 0.001 | 58, 0.077 | [3.254; 1.232-8.591], 2.5, 0.012* |
| 86 | 142 | 39 | R3 | -.399**, 0.006 | 68, 0.012* | [2.903; 1.17-7.2], 3.47, 0.016* |
| 87 | 155 | 43 | R3, qMSP | -.334*, 0.023 | 68, 0.024* | [1.786; 0.793-4.025], 2.41, 0.156 |
| 88 | 160 | 24 | R3, qMSP | -0.162, 0.283 | 52, 0.217 | [1.837; 0.638-5.289], 1.39, 0.252 |
| 89 | 172 | 54 | R3, qMSP | -.338*, 0.021 | 71, 0.011* | [2.255; 1.071-4.748], 1.73, 0.028* |
| 90 | 188 | 22 | R3 | -0.079, 0.603 | 39, 0.527 | [1.662; 0.578-4.781], 1.39, 0.341 |
| 91 | 199 | 48 | R3 | -.313*, 0.034 | 52, 0.756 | [1.789; 0.841-3.804], 1.45, 0.126 |
| 92 | 202 | 26 | R3 | -0.282, 0.058 | 55, 0.132 | [1.36; 0.581-3.183], 1.33, 0.476 |
| 93 | 205 | 33 | R3 | -.347*, 0.018 | 58, 0.077 | [2.413; 1.061-5.49], 2.57, 0.031* |
| 94 | 217 | 20 | R3 | -0.183, 0.225 | 45, 0.546 | [6.257; 0.847-46.244], 3.33, 0.04* |
| 95 | 238 | 33 | R3 | -0.164, 0.277 | 65, 0.024* | [2.289; 0.923-5.68], 2.39, 0.067 |
| 96 | 242 | 28 | R3 | -.430**, 0.003 | 48, 0.694 | [1.379; 0.63-3.02], 1.4, 0.419 |
| 97 | 255 | 39 | R3 | -0.219, 0.143 | 55, 0.332 | [1.677; 0.765-3.678], 1.45, 0.192 |

**ρ** = Spearman’s rank correlation coefficient

* p-value < 0.05

** p-value < 0.01

Seven highlighted CpG sites are significantly correlated with all three; MGMT mRNA expression, protein expression and progression-free survival.

**NA**: These CpG sites were either methylated CpGs 1 to 6) or unmethylated CpGs 51 to 70) in majority of samples leaving them unsuitable for significance testing.
