# Supplementary material for: Comprehensive Analysis of MGMT Promoter Methylation: Correlation with MGMT Expression and Clinical Response in GBM
Source: PLoS One. 2011 Jan 7;6(1):e16146. doi: 10.1371/journal.pone.0016146 (PMC3017549; doi:10.1371/journal.pone.0016146)
Supplement: Table S2 — Correlation of MGMT promoter methylation patterns with MGMT mRNA expression, MGMT protein expression and PFS. (DOC) [file pone.0016146.s005.doc]

Table S2 Correlation of MGMT promoter methylation patterns with MGMT mRNA expression, MGMT protein expression and PFS.

| **Classification** | **mRNA ρ, p-value)** | **Protein %concordance, p-value chi square)** | **PFS hazard ratio; 95% confidence interval; Median ratio, p-value)** |
| --- | --- | --- | --- |
| R1 | -.382, 0.009** | 55, 0.667 | 2.034; [0.964-4.29], 1.67, 0.057 |
| R2 | -.373, 0.011* | 74, 0.001** | 2.275; [1.025-5.052], 1.81, 0.039* |
| R3 | -.321, 0.029* | 71, 0.006** | 2.71; [1.243-5.908], 2.57, 0.009** |
| 3R | -.424, 0.003** | 74, 0.003** | 5.23; [2.089-13.097], 2.57, 0** |
| MLPA | -.545,0.000001** | 71, 0.006** | 3.076; [1.301-7.27], 2.57, 0.007** |
| qMSP | -.459, 0.001** | 71, 0.002** | 1.707; [0.728-4.003], 1.67, 0.213 |

* Significant correlations p-value < 0.05).

** Significant correlations p-value < 0.01).
